# *JAK2* and Beyond: *JAK2*V617 Mutational Study of Myeloproliferative Disorders and Haematological Malignancies

**DOI:** 10.31557/APJCP.2019.20.12.3611

**Published:** 2019

**Authors:** Nidda Syeed

**Affiliations:** 1 *College of Applied Medical Sciences, Taibah University, Madinah Saudi Arabia, *; 2 *Department of Immunology and Molecular Medicine, Sher-I-Kashmir Institute of Medical Sciences, Srinagar, Kashmir, 190011, India. *

**Keywords:** Myeloproliferative disorders (MPD’s), Kashmir, acute myeloid leukemia (AML), Polycythemia Vera(PV)

## Abstract

**Background:**

Janus Tyrosine Kinase-2 (*JAK2* V617F), a novel point mutation affecting the MPD’S is a somatic gain-of-function mutation. It alters a highly conserved amino acid valine in the negative regulatory JH2 domain to phenylalanine predicted to dysregulate kinase activity.

**Aim:**

To evaluate the prevalence and clinical significance of *JAK2* V617F mutation in various MPD’s as well as in hematological malignancies.

**Subjects and Methods:**

*JAK2* mutation was assessed in 90 patients with myeloproliferative disorders and 47 leukemic patients. In addition, peripheral blood samples from 90 healthy donors were also collected as control. We used a highly sensitive Allele-Specific polymerase chain reaction (AS-PCR) for the detection and confirmed the mutation further by direct sequencing.

**Results:**

Our results showed significant differences between various disorders with respect to either the proportion of positivity or that of mutant alleles. *JAK2*-V617F was detected in 67/90 MPD patients and 02/17 for AML,01/11 for ALL-L1,02/12 for ALL-L2 and 02/07 for CML and 90 healthy controls.

**Conclusion:**

From the above findings it is evident that the *JAK2* V617F mutation is widespread not only in MPD’s but also in hematological malignancies, which might as well lead to the new classification of MPD’S. Our data also suggest that different genetic events may lead to JAK-STAT pathway activation in different malignancies.

## Introduction

The myeloproliferative disorders are a heterogeneous group of diseases characterized by excessive production of blood cells by hematopoietic precursors (Bench et al., 2001). The MPDs constitute a subcategory of CMD and include CML, ET, PV, and IMF (Dameshek, 1951). These four clinicopathologic entities were described between 1845 and 1934 and it was the substantial overlap in their clinical and laboratory manifestations that inspired William Dameshek to group them together as “MPD” in 1951 (Dameshek, 1951). The initial classification of the MPDs was based on clinical, morphological and biological criteria (Bench et al., 2001). In most instances, the molecular pathogenesis of the MPDs was identified by the examination of recurrent chromosomal translocations (i.e., CML or atypical CML) or as the consequence of a clinical response to tyrosine kinase inhibitors. By contrast, recurrent chromosomal translocations were not found in patients with PV, ET and IMF. It has recently been discovered that a single-site, clonal, gain-of-function mutation of the tyrosine kinase, *JAK2* (*JAK2* V617F) is present in myeloid cells from the majority of patients with chronic myeloproliferative disorders (CMDs) (Goldman, 2005; Kaushansky, 2005).

JAK2 V617F -is a somatic gain-of-function mutation in MPD’S (Kralovics et al., 2005). It alters a highly conserved amino acid valine in the negative regulatory JH2 domain to phenylalanine predicted to dysregulate kinase activity (Saharinen et al., 2003). Chromosomal translocations resulting in fusions deregulating *JAK2* activity are implicated in leukemias as well (Peeters et al., 1997; Nunez et al., 2003; Reiter et al., 2005).

Discovery of the *JAK2* V617F mutation has added a new dimension to the overall understanding of the myeloproliferative disorders (Baxter et al., 2005; Sandberg, 2004). *JAK2* is a cytoplasmic protein-tyrosine kinase that catalyzes the transfer of the gamma-phosphate group of adenosine triphosphate to the hydroxyl groups of specific tyrosine residues in signal transduction molecules. The main downstream effectors of *JAK2* are a family of transcription factors known as signal transducers and activators of transcription (STAT) proteins. Although *JAK2* V617F expression does not define the limits of these disorders, it does permit the clinical identification of a significant proportion of them. Several lines of evidence suggested that *JAK2* was the most likely candidate gene involved in the pathogenesis of PV (Cross and Reiter, 2002). The *JAK2* V617F mutation in the pseudokinase autoinhibitory domain results in constitutive kinase activity (Levine et al., 2005 ; Saharinen,2 000) and induces cytokine hypersensitivity or independence of factor-dependent cell lines. The V617F mutation frees the *JAK2* kinase from the control of its autoregulatory domain resulting in constitutive activation in the absence of a physiologic signal, the V617F region plays a direct role in negatively regulating *JAK2* signaling (Saharinen et al., 2003). *JAK2* V617F mutation was observed in a majority of PV (Prchal, 2005) patients and a significant proportion of patients with other MPDs, including ET, IMF and some rare MPDs (Levine et al., 2005; James, 2005). The widespread presence of *JAK2* V617F in MPDs suggests that it may contribute to the pathogenesis of these diseases (Jones et al., 2005). However, it is not clear whether *JAK2* V617F can be implicated as the direct and primary cause of PV, ET, or other MPD’S, nor is the relationship between the different MPDs that share the *JAK2* mutation understood. Here, we investigated the presence of *JAK2* V617F in different MPD patients and our results suggest that *JAK2* V617F is present in PV, ET, as well as in hematological malignancies of AML, CML and ALL (L1-L2). Sequencing of genomic DNA from granulocytes has been used in most studies to detect the mutation (Zhao et al., 2005), however, our study primarily depended on sensitive technique of mutation detection using AS-PCR assay, followed by direct sequencing.

*Patients and Controls*

*JAK2* mutation was assessed in 90 patients with myeloproliferative disorders from Kashmir valley, the northern most part of India located at 30,000 ft above sea level. The study included patients of Polycythemia Vera (PV), Essential Thrombocythemia (ET), Chronic Myeloid Leukaemia (CML), Acute Myeloid Leukaemia (AML) and Acute Lymphoblastic Leukaemia (ALL). Blood samples were collected from the department of Clinical Haematology, Sher-I-Kashmir Institute of Medical Sciences, Srinagar, Kashmir. The study was approved by the Institutional Ethics Committee. In addition, peripheral blood samples from 90 healthy donors were also collected as control. Only patients with confirmed diagnosis were included. 

## Materials and Methods

Allele Specific PCR (AS-PCR ) was performed using a common reverse primer *JAK2* R (5’-CTGAATAGTCCTACAGTGTTTTCAGTTTCA-3’) and 2 forward primers named *JAK2* F Mut (5’-AGCATTTGGTTTTAAATTATGGAGTATATT-3’)specific for the mutant allele containing an intentional mismatch at third nucleotide from the 3’ end and *JAK2* F WT (5’-ATCTATAGTCATGCTGAAAGTAGGAGAAAG-3’) as internal control which amplifies the wild-type allele. PCR was performed, after an initial denaturation of 1 min to 95°C, 35 cycles of denaturation, annealing and extension of 1 min each with the temperatures of 95, 58 and 72°C respectively. The PCR amplified products were run on 6% polyacrylamide gel along with 100 bp ladder, the gels were stained by silver nitrate, Direct sequencing of PCR products was performed in randomly selected patients.

*Patients’ characteristics*

Patients of different ages with pathologically proven myeloproliferative disorders were analyzed for *JAK2*-positive mutation, 53 patients were diagnosed with polycythemia vera (PV), 37 patients were diagnosed with essential thrombocythemia(ET), 17 patients with acute myelocytic leukemia (AML), 11 with acute lymphoblastic leukemia (ALL-L1),12 with acute lymphoblastic leukemia (ALL-L2),07 with chronic myelocytic leukemia (CML). Following the recent report of the *JAK2* V617F mutation in patients with PV, ET, and IMF, (Baxter et al., 2005; Kralovics et al., 2005; Levine et al., 2005; James et al., 2005) we focused specifically on this abnormality. Consistent with this observation, a significantly lower platelet count has been shown in *JAK2*V617F-positive patients, as opposed to mutation-negative, patients with ET (Antonioli et al., 2005 ; Beutler and Waalen, 2005).

The diagnosis of patients was made using the following diagnostic criteria: (i) hemoglobin >185 ug/dl in males or >165 ug/dl in females. (ii) Palpable or radiological splenomegaly. (iii) Platelet count >400 × 10^3^/ul or white blood cells >12 × 10^3^/ul. Reference data on hematological values from other sources are somewhat similar (Beutler, 2005). The patient details and the results are shown in Table 1 and Table 2. 

*Diagnostic Criteria for Haematological malignancies*

The diagnosis and classification of AML, ALL and CML was performed by a qualified hematopathologist. The presence of certain morphological and clinical features were used to diagnose AML, ALL, CML patients. The first clue to a diagnosis of AML, ALL, CML was typically due to abnormal result on a complete blood count. Laboratory tests were done which showed abnormalities including blood count tests, renal function tests, electrolyte tests and liver enzyme tests. We performed bone marrow examination to identify the type of abnormal blood cells, marrow was examined via light microscopy and flow cytometry using specific CD markers for AML (CD33, CD65, CD117),ALL (CD19, CD20, CD22, CD10) and the most important criteria for CML is the presence of Ph+ chromosome, which was done by genetic analysis for BCR-ABL fusion gene. Cytochemical stains on blood and bone marrow smears were used for distinction of AML, ALL and CML. The combination of a myeloperoxidase or Sudan black stain and a non - specific esterase stain provided the desired information in most cases. The non-specific esterase stain was used to identify a monocytic component in AMLs and to distinguish a poorly differentiated monoblastic leukemia from ALL (Vardiman et al., 2002). 

**Figure 1:**
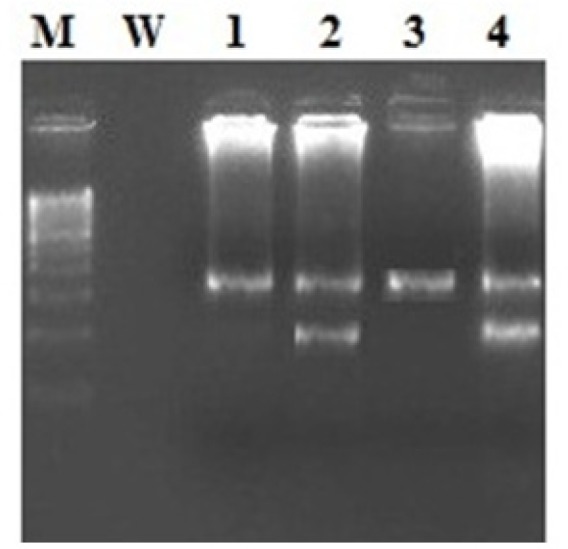
Gel Picture Showing the Amplified Allele Specific PCR Product Showing the Wild Band of 364bp and a Mutant Band of 203bp. LANE M, DNA Ladder; LANE W, Water; LANE 1, P1; LANE 2, P2; LANE 3, P3; LANE 4, P4; Lane 2,4 Showed the Mutant Allele Having Two Bands mutant 203 and wild 364 bp; Whereas Lane 1,3 showed the wild type carrying one Single band of 364 bp

**Table 1:** Patient Details and Summary of Results of Polycythemia Vera and Essential Thrombocythemia

Disease Type	No. of Patients	Positive patients (%age)	Median Age (range)	Haemoglobin (Hb g/l)	Platalet count (×10^3^/µL)	Palpable Splenomegaly
PV	53	53/53 (100%)	45-62	16-21	440-775(×10^3^/uL)	14/30
ET	37	35/37 (92.31%)	40-60	13-15	440-898(×10^3^/uL)	06/13

**Table 2:** Patient Details and Summary of Results of Leukemic Patients

Disease Type	No. of Patients	Positive patients (%age)	Median Age (range)	Haemoglobin (Hb g/l)	Platalet count (×10^3^/µL)	Palpable Splenomegaly
AML	17	02 (11.76%)	15-30	9-12	33-150 (×10^3^/µL)	09/17
ALL-L1	11	01 (9.09 %)	12-30	8-11	47-174 (×10^3^/µL)	05/11
ALL-L2	12	02 (16.6%)	11-28	8-11	44-170 (×10^3^/µL)	08/12
CML	7	02 (28.57%)	35-45	9-13	110-838 (×10^3^/µL)	03/07

## Results

*Incidence of Jak2 (V617F) mutation*

*JAK2* 1849G>T mutation status was analyzed by allele specific polymerase chain reaction (AS-PCR) in 90 samples of patients with myeloproliferative disorders as well as hematological malignancies. 53 Polycythemia Vera patients, 37 Essential thrombocythemia patients, 47 leukemic patients, and 90 healthy controls. The mutated *JAK2* allele differs from the wild-type *JAK2* allele by just one nucleotide exchange (G>T) leading to the valine to phenylalanine (V>F) transition. For this study, we designed an allele-specific PCR (AS-PCR). The technique of AS-PCR, one of the commonly used mutation detection methods, is applicable both for the qualitative and quantitative measurement of *JAK2* V617F (Beutler and Waalen, 2006). Our analysis of various myeloproliferative disorders and hematologic malignancies confirmed the high prevalence of *JAK2* mutation in PV ( [100%] 53 of 53 patients) and ET ([92.31%] 35 of 37 patients), and significant prevalence in hematological malignancies including, AML sub-types (M1, M2) ([11.76%] 02 of 17 patients), ALL-L1 ( [9.09%] 01 of 11 patients, and ALL-L2 ( [16.6%] 02 of 12 patients) and CML ( [28.57%] 02 of 07 patients).Patient details and summary of results are given in the table 1.Furthermore the mutation was not detected in 90 healthy controls. In patients of PV and ET *JAK2* V617F mutation has been associated with advanced age mostly above 40 years, high hemoglobin, high platelet count and marked palpable splenomegaly.

Overall, our results confirm previous reports on the prevalence of *JAK2* mutations in PV, ET, CML, and MDS (Jones et al., 2005) and suggests that the *JAK2* V617F mutation is common in MPD’S as well in hematological disorders, although the occurrence of *JAK2* V617F mutation in leukemic patients is less compared to PV and ET (Jones et al., 2005; Steensma et al., 2005). In our study, the incidence of *JAK2* V617F mutation was not similar to other publications differing in the occurrence of *V617F* mutation in AML, ALL cases (Peeters et al., 1997).This observation opens new avenues for fundamental and clinical researches and has direct implications for the diagnosis .

We have identified the *JAK2* V617F mutation in 90 patients with MPD’S, as well as a significant percentage of this mutation in the hematological malignancies (AML, ALL, CML). *JAK2* V617F (Val617Phe) G>T mutation, was recently described in most patients with polycythemia Vera (PV) and in essential thrombocythemia (ET) and mutation of both *JAK2* alleles has been reported in approximately 100% of the PV patients (Zhao et al., 2005).

## Discussion

*JAK2* is a PTK involved in signaling pathways by members of the single chain receptors (e.g. EPOR, TPOR, GHR, PRLR), the IL-3 receptor family (IL-3R, IL-5R and GM-CSF-R), the gp130 receptor family and the class II receptor cytokine family (Kisseleva et al., 2002; Darnell et al., 1994). The JAK family PTKs have a unique domain structure with a kinase domain (JH1) adjacent to a pseudokinase domain (JH2) (Darnell et al., 1994; Ihle, 1995). The JH2 domain lacks essential amino acid residues conserved in active protein kinases. The third Gly of the canonical GXGXXG motif in subdomain I is replaced by Thr; the conserved Asp in the DxxxxN motif in subdomain VIB is replaced by Ala; DFG in subdomain VII becomes DPG. The lack of these key residues would make the pseudokinase domain catalytically inactive. It has been postulated that V617, C618, and other local residues inhibit movement of the activation loop from its inactive to its active conformation (ie, the V617 region plays a direct role in negatively regulating *JAK2* signaling (Lindauer et al., 2001). Infrequent occurrence of this unique *JAK2* mutation has been reported recently in chronic myeloid leukemia (CML), acute myelocytic leukemia, and acute lymphoblastic leukemia (Jones et al., 2005). The discovery of the *JAK2* V617F mutation is a major advance in enhancing our understanding of both the molecular pathogenesis and the clinical aspects of PV and several other MPDs. Although there are strict diagnostic criteria for MPD subtypes, precise categorization remains a subject of debate (Thiele and Kvasnicka, 2003). This observation opens new avenues for fundamental and clinical researches and has direct implications for the diagnosis and classification of MPDs. Our research focuses on MPDs (PV, ET) and certain leukemia’s (AML, ALL, CML) for *JAK2* V617F mutation, which might constitute a distinctive entity.

*JAK2* has become a potential target for developing therapeutic drugs to treat these diseases. Our data suggest the prevalence *JAK2* mutation in other hematological disorders including CML, AML, and ALL. Analysis of patients with acute myeloid leukemia (AML) sub types in other studies has shown that *JAK2* V617F is present in a low proportion of patients (Jelinek et al., 2005). In contrast, the prevalence of *JAK2* V617F in ALL has been reported to be low compared to AML cases; however, data on the frequency of *JAK2* V617F in ALL, AML subtypes are limited. Our data shows that the *JAK2*/STAT5 signal transduction pathway is constitutively activated in megakaryocytic leukemic patients that prompted us to analyze the patients with AML, ALL and CML. Furthermore our analysis of various hematologic malignancies confirmed the significant prevalence of *JAK2* mutation in AML, ALL, CML patients, contradictory to other studies that suggests *JAK2* V617F is less common in leukemic patients. (Levine et al., 2005; Jelinek et al., 2005). It is known that the clinical diagnostic precision varies between clinicians and it is possible therefore that the true incidence of V617F in PV and IMF is somewhat higher than we found in this study. 

Our current data further strengthens the fact that our ethnic Kashmiri population has some unique mutational characteristics in terms of pure genetic pool due to consagnious marriages, different dietary habits and lifestyle. which is not been profoundly seen in other populations studied; In Breast cancers, authors found very low percentage of mutations in BRCA1 gene but much higher percentages in *TP53* gene (Hussain et al., 2009),Similarly we have a unique cancer called Kangri cancer of Skin where we showed different mutational spectrum in TP53 and *PTEN* genes as compared to other skin cancers. These findings might justify our present observations of higher percentages of *JAK2* mutations in our patient group. We believe that *JAK2* mutation testing will rapidly become a frontline test for individuals with a suspected diagnosis of an MPD and the same genetic event can play a role in the pathogenesis of a wide spectrum of myeloid and lymphoid malignancies. We believe these observations warrant a comprehensive search for activated tyrosine kinases in MPD’S and hematological malignancies, as there are likely additional unidentified genetic events with biological and therapeutic significance. Additional in vitro and in vivo studies are needed to determine the cause of the specificity of *JAK2* V617F for myeloid and lymphoid diseases, as second mutations, host modifiers, differential cytokine receptor expression, and other factors may influence the ultimate phenotype of hematopoietic progenitors that acquire the *JAK2* V617F mutation. Our data also suggest that different genetic events may lead to JAK-STAT pathway activation in different malignancies.
